# The impact of digital teaching on learning burnout in college students: the moderating role of self-efficacy and the mediating effect of learning adaptability

**DOI:** 10.1186/s12909-025-08330-0

**Published:** 2026-01-07

**Authors:** Mingyuan Huang, Shuwen Wang, Wen Yao, Zhengjing Tan, Hongyu Zhao, Weiping Chen

**Affiliations:** 1 Department of Health Inspection and Quarantine, School of Public Health, Guangdong Medical University, Dongguan, 523808 China; 2 The Second Clinical School, Guangdong Medical University, Dongguan, China; 3 School of Nursing, Guangdong Medical University, No. 1, Xincheng Avenue, Songshan Lake Science Park, Dongguan, 523808 China

**Keywords:** Digital learning, Learning burnout, Learning adaptability, Self-efficacy, Longitudinal study

## Abstract

**Introduction:**

The rapid adoption of digital teaching has transformed higher education, offering both flexibility and accessibility, but also raising concerns about its potential impact on the mental health of students and learning burnout. In this study, we investigated the potential effect of digital teaching on learning burnout in college students, with specific focus on the mediating role of learning adaptability and the moderating role of self-efficacy.

**Methods:**

The study population was selected using cluster sampling from the entire cohort of first-year medical students, resulting in a sample of 529 participants. Respondents completed a questionnaire monthly throughout the 12-month follow-up period to analyze the transition from traditional to digital teaching. Regression analysis was applied to investigate the impact of digital teaching on learning burnout, as well as the mediating role of learning adaptability and the moderating role of self-efficacy. Data were collected every month, including self-reported measures relating to burnout, adaptability and self-efficacy.

**Results:**

Digital teaching significantly increased learning burnout (regression coefficient: 0.042, *P* < 0.01); the contributing factors including increased cognitive demands and reduced social interactions. Learning adaptability mediated this relationship, as higher adaptability mitigated burnout (-1.835, *P* < 0.01). Finally, self-efficacy moderated the relationship between digital teaching and burnout; students with higher levels of self-efficacy experienced less burnout (-0.062, *P* < 0.05).

**Conclusion:**

Digital teaching presents both challenges and opportunities, increasing learning burnout while fostering adaptability. Enhancing learning adaptability and self-efficacy can mitigate the adverse effects of digital teaching. Improvements in students’ reduced learning ability, decreased learning efficiency, and lack of interest in learning (-1.835). These findings suggest that educators and policymakers should modularize the curriculum and incrementally design instructional tasks. These methods are expected to enhance students’ self-efficacy and sense of mastery, with the goal of preventing learning burnout in digital environments.

**Supplementary Information:**

The online version contains supplementary material available at 10.1186/s12909-025-08330-0.

## Introduction

Over recent years, the rapid integration of digital teaching methods, including online and hybrid learning formats, has fundamentally reshaped higher education [1]. While this transformation has been underway for some time, the onset of the COVID-19 pandemic accelerated its global adoption, compelling educational institutions to swiftly adapt to remote learning modalities [2]. Digital teaching has garnered significant praise for its numerous advantages, including greater accessibility, flexibility and the potential for personalized learning experiences. However, alongside these benefits, significant concerns have emerged regarding the impact of digital learning on the mental health, engagement, and academic performance of students [3]. A particularly concerning issue is the increasing prevalence of learning burnout among college students navigating the complexities of digital education [4]. Learning burnout is a multifaceted psychological condition characterized by emotional exhaustion, depersonalization, and a diminished sense of accomplishment in academic endeavors [5]. This condition manifests through a range of symptoms, including chronic fatigue, reduced motivation and feelings of detachment from academic tasks [6]. Importantly, learning burnout has been associated with detrimental outcomes, including lower academic performance, increased stress and a higher likelihood of dropping out of educational programs [7]. The digital learning environment introduces unique challenges that exacerbate the risk of burnout, including prolonged screen time, feelings of social isolation, technical difficulties and the increased cognitive demands of navigating digital platforms [8]. Overcoming difficulties is a crucial factor in learning adaptation. Learning adaptability primarily enables students to maintain consistency between their learning and the external environment by overcoming challenges, thereby fostering a tendency to achieve learning efficacy [9]. At the same time, self-efficacy makes students more likely to persevere in their studies, continuing to persevere even when faced with academic challenges or difficulties [10].

While the extant literature provides valuable insights into the individual constructs of digital teaching, learning burnout, self-efficacy and learning adaptability, there is a notable scarcity of their interconnected dynamics [11, 12]. Previous studies have examined contributing factors to learning burnout, such as academic stress and inadequate social support, along with the protective roles of self-efficacy in promoting engagement and learning adaptability in facilitating adjustment to digital environments [13–15]. However, few studies have systematically integrated these constructs to investigate their collective influence on learning burnout. Moreover, existing research tends to focus on either the psychological effects of digital teaching or the individual traits that moderate these effects, without considering how these traits might interact with the learning environment to shape the experiences of students to burnout [16]. For example, while some studies have highlighted the negative impact of digital teaching on the mental health of students [17, 18], others have emphasized the protective role of self-efficacy in online learning settings [19, 20].

The primary objective of this study was to investigate the impact of digital teaching on learning burnout in college students, with a specific focus on the moderating role of self-efficacy and the mediating effect of learning adaptability. By identifying the protective roles of self-efficacy and learning adaptability, this research can inform the development of targeted interventions aimed at enhancing these traits among students. For example, institutions could implement training programs to foster self-efficacy or design curricula that promote adaptability through experiential learning activities [21].

### Self-efficacy and digital teaching

Self-efficacy, a central construct in Bandura’s Social Cognitive Theory, refers to an individual’s belief in their ability to successfully perform a specific task or achieve a desired outcome [14, 22]. For students’ psychological processes, regardless of their form, they will alter the level and intensity of self-efficacy [23]. This construct is particularly important in educational settings as it can influence the motivation, persistence, and resilience of students in the face of academic demands. Digital teaching often involves asynchronous schedules, minimal face-to-face interaction, and increased reliance on self-regulation; these factors require students to adapt to a more independent mode of learning [24, 25]. Students with high levels of self-efficacy are better equipped to navigate these challenges, perceiving them as opportunities to enhance their skills and problem-solving capabilities. For instance, these students are more likely to engage in proactive behaviors, such as seeking help from a companion when needed, utilizing technological tools effectively, and maintaining motivation despite setbacks [10, 26, 27]. This proactive approach not only enhances learning outcomes but also reduces the likelihood of stress and burnout. Conversely, students with low levels of self-efficacy are more vulnerable to the inherent stressors of digital learning environments. Previous research suggested that these students are more likely to view challenges, such as technical difficulties or the lack of immediate feedback, as insurmountable barriers [28, 29]. This perception can lead to feelings of frustration, helplessness and disengagement; these are precursors to learning burnout [30]. Learning burnout, characterized by emotional exhaustion, depersonalization, and a reduced sense of accomplishment, is more prevalent among students with low levels of self-efficacy due to their limited ability to cope with stressors in an effective manner [31].

Numerous studies have underscored the critical role of self-efficacy in mitigating against the negative effects of digital teaching. Research indicates that students with higher levels of self-efficacy are more likely to employ adaptive coping strategies, such as time management and goal setting, thus mitigating the adverse impacts of online learning [32]. Furthermore, self-efficacy positively correlated with the satisfaction and performance of students in digital learning environments, thus suggesting that confidence in our own abilities can foster engagement and reduce stress [19]. Moreover, self-efficacy has been identified as a key Maslach predictor of academic resilience. Academic resilience refers to the ability of students to persevere and succeed despite significant stressors or challenges. In the context of digital learning, resilience often manifests as a student’s ability to troubleshoot technical problems, adapt to new learning platforms, and maintain consistent effort in self-directed tasks.

In addition to its direct effects, self-efficacy also moderates the relationship between digital teaching and learning burnout. This moderating effect is particularly significant in the context of digital education where the demands for self-regulation and autonomy are higher than in traditional classroom settings. For instance, students with high levels of self-efficacy are less likely to perceive digital teaching as overwhelming, even when faced with isolation, increased workload, or technical challenges. Their belief in their own abilities allows them to maintain focus and employ adaptive strategies, such as breaking tasks into manageable components or seeking support from instructors and peers. In contrast, students with low levels of self-efficacy are more likely to experience enhanced levels of stress and frustration as they may lack the confidence to effectively manage the demands of digital learning. This stress can be further compounded by the absence of immediate social support, which can exacerbate feelings of isolation and helplessness. Consequently, these students are more susceptible to learning burnout, as they are less able to cope with the cumulative pressures of digital education.

### Learning adaptability: a mediating factor

Learning adaptability refers to an individual’s capacity to modify their learning strategies, behaviors and attitudes in response to changing demands or environmental factors [33]. In educational contexts, adaptability is a critical skill that enables students to navigate challenges in an effective manner, such as transitioning between different learning formats or coping with unexpected disruptions. The concept of learning adaptability encompasses several dimensions, including cognitive flexibility, emotional regulation, and behavioral adjustment, all of which are crucial for success in dynamic learning environments.

Learning adaptability is particularly important in the context of digital teaching in which the learning environment is characterized by self-directed schedules, limited face-to-face interaction, and a heavy reliance on technology. While these features provide flexibility, they also place significant demands on the ability of students to self-regulate, manage time effectively, and seek support independently. Students with high levels of learning adaptability are better equipped to meet these demands as they can adjust their strategies and behaviors to align with the unique requirements of digital education [8, 34]. For instance, students may employ proactive time management techniques, develop problem-solving skills to address technical issues, and seek virtual collaboration with peers to maintain engagement.

Conversely, students with low levels of learning adaptability often struggle to cope with the demands of digital learning and find it difficult to organize their study schedules, stay motivated, or utilize digital platforms effectively. This lack of adaptability can lead to feelings of frustration, disengagement, and increased stress; all of these factors can contribute to learning burnout, characterized by emotional exhaustion, depersonalization, and reduced academic efficacy.

#### Empirical evidence for learning adaptability

Previous research has demonstrated the pivotal role of learning adaptability in mitigating the negative effects of digital teaching. For example, Kuss et al. [35] demonstrated that students with high levels of adaptability are more likely to perceive digital learning challenges as manageable and even stimulating. By adopting flexible thinking and behaviors, these students can transform obstacles into opportunities for growth, thus reducing the risk of burnout. Other studies have shown that adaptability can enhance the ability of students to cope with the cognitive demands of digital learning, such as processing large volumes of online information or transitioning between multiple digital tools [36].

Another key dimension of learning adaptability is behavioral adjustment. This involves the modification of study habits, adopting new learning strategies, and experimenting with different approaches to problem-solving. Previous research demonstrated that students who exhibit high levels of behavioral adaptability are more likely to remain engaged in their studies, even when facing significant challenges. This level engagement not only improves academic performance but also serves as a protective factor against burnout [4].

#### The mediating role of learning adaptability

Learning adaptability plays a critical mediating role in the relationship between digital teaching and learning burnout. Digital teaching, with its unique challenges, has the potential to either foster or hinder adaptability, depending on the support provided and the individual characteristics of students. For students with high levels of adaptability, digital teaching can serve as a platform for developing new skills and strategies, thus enhancing their resilience and reducing the risk of burnout. These students are more likely to view the demands of digital teaching as opportunities to grow and expand their capabilities.

In contrast, students with low levels of adaptability may perceive digital teaching as both overwhelming and unmanageable. The absence of direct guidance and the need for self-regulation can exacerbate their stress levels, thus leading to an enhanced risk of burnout. However, by improving adaptability through targeted interventions, educators can help students to bridge this gap and transform their learning experiences. For example, teaching students how to set realistic goals, manage their time effectively, and navigate digital tools can significantly enhance their adaptability, thereby reducing the negative effects of digital teaching on burnout [37].

The mediating effect of adaptability is supported by empirical findings. Previous studies have demonstrated that adaptability enables students to manage the stressors of digital learning more effectively, such as by employing cognitive and emotional coping strategies [32]. Moreover, adaptable students are more likely to develop a sense of control and agency; these factors are essential for maintaining engagement and motivation in digital learning environments.

### Digital teaching and learning burnout: an integrative framework

Digital teaching, encompassing online, hybrid, and technology-mediated instruction, has transformed the educational landscape by providing unprecedented levels of accessibility and flexibility. Students can now learn from a diverse range of geographic locations, engage with personalized content, and balance education with other responsibilities. Despite these advantages, digital teaching presents unique challenges that can have profound implications for the mental health and academic performance of students.

One of the most significant challenges associated with digital teaching is learning burnout, a psychological condition characterized by emotional exhaustion, depersonalization, and a reduced sense of accomplishment [38]. Learning burnout arises when students face prolonged stress and an inability to cope with academic demands effectively. In digital learning environments, where students must navigate asynchronous schedules, manage their time independently, and contend with technological disruptions, the risk of burnout is enhanced. These environments often blur the boundaries between academic and personal life, contributing to chronic stress and reducing opportunities for recovery [39].

#### The unique demands of digital teaching

Digital teaching introduces several stressors that differ significantly from those of traditional classroom settings. First, the absence of direct, face-to-face interaction with instructors and peers can lead to feelings of isolation and reduced social support. Social interaction is a critical factor in fostering a sense of belonging and engagement in education; its absence in digital teaching can erode a student’s motivation and exacerbate feelings of detachment [35]. Second, digital teaching places heavy emphasis on self-regulation. Students are expected to manage their schedules, prioritize tasks, and stay motivated without the external structure provided by a physical classroom. While these demands foster autonomy and independence for some, they can overwhelm others, particularly those with lower levels of self-efficacy or adaptability [40]. The cognitive and emotional strain of balancing academic responsibilities with personal life can lead to exhaustion, frustration, and disengagement.

Third, technological challenges are a pervasive source of stress in digital learning. From unstable internet connections to unfamiliar learning platforms, these disruptions can hinder the ability of students to focus and achieve their academic goals. For students who lack the necessary technical skills or resources, these challenges can feel insurmountable, increasing the likelihood of burnout [41].

#### Protective factors: self-efficacy and learning adaptability

Despite these challenges, individual differences in self-efficacy and learning adaptability can buffer the negative effects of digital teaching and reduce the risk of burnout. Self-efficacy, as discussed in section "Sample Selection", empowers students to approach challenges with confidence, enabling them to persevere and adopt effective coping strategies. Similarly, learning adaptability, elaborated in section "Inclusion and Exclusion Criteria", equips students with the flexibility to adjust their behaviors and strategies in response to changing academic demands. Students with high levels of self-efficacy and adaptability are more likely to perceive the demands of digital teaching as manageable and even rewarding. These students exhibit greater resilience, using their skills to navigate technical difficulties, maintain motivation and seek support when needed. In contrast, students with low levels of self-efficacy and adaptability are more likely to view digital teaching as overwhelming, amplifying stress and increasing their susceptibility to burnout.

Empirical evidence supports the moderating and mediating roles of self-efficacy and adaptability in the relationship between digital teaching and burnout. For example, Artino [19] found that students with higher levels of self-efficacy reported greater satisfaction and engagement in online courses, while Li et al. [36] demonstrated that adaptability enhances the ability of students to cope with the demands of digital learning.

#### Integrating self-efficacy, learning adaptability and burnout

Our integrative framework proposes that the impact of digital teaching on learning burnout is not uniform but depends on the interplay between digital teaching, self-efficacy, and learning adaptability. In particular, we propose that self-efficacy moderates the relationship between digital teaching and burnout by shaping how students perceive and respond to challenges. Students with high levels of self-efficacy are more likely to use adaptive coping strategies, thus reducing the stress associated with digital teaching. We also propose that learning adaptability mediates this relationship by enabling students to adjust their behaviors and strategies, thus transforming the challenges of digital teaching into opportunities for growth. This framework emphasizes the dual role of digital teaching: while this form of teaching introduces new stressors that increase the risk of burnout, it can also provide opportunities to develop critical skills, such as adaptability and self-regulation. By fostering these skills, digital teaching can empower students to succeed in dynamic learning environments.

In this study, we investigated the complex interplay between digital teaching, self-efficacy and learning adaptability in the context of learning burnout. By integrating theoretical insights and empirical evidence, our study provides a robust framework for understanding how differences between individuals mediate and moderate the effects of digital teaching. The findings of this study hold significant implications for educators and policymakers, offering actionable strategies to enhance student resilience and well-being in the digital age.

Based on the theoretical and empirical insights discussed above, we propose the following three hypotheses:

- H1: Digital teaching has a direct positive effect on learning burnout in college students.
- The unique stressors of digital learning, such as asynchronous schedules, reduced social interaction, and technical difficulties, contribute to increased levels of burnout in students. These stressors place additional cognitive and emotional demands on students, particularly those who struggle with self-regulation and technological adaptation.
- H2: Self-efficacy moderates the relationship between digital teaching and learning burnout, such that the positive effect of digital teaching on learning burnout is weaker for students with high levels of self-efficacy than those with low self-efficacy. This hypothesis reflects the protective role of self-efficacy in mitigating the negative effects of digital teaching. Students with higher levels of self-efficacy are better equipped to manage the challenges of online learning and are less likely to experience burnout.
- H3: Learning adaptability mediates the relationship between digital teaching and learning burnout, such that digital teaching enhances students’ learning adaptability, which in turn reduces learning burnout. This hypothesis reflects the dual role of digital teaching in both challenging students and fostering their adaptability. By enhancing adaptability, digital teaching can empower students to overcome obstacles and thrive in dynamic learning environments, ultimately reducing the risk of burnout.

## Materials and methods

### Sample selection

Subjects were first-year medical students at a top-tier university in Guangdong Province, China. Prior to September 2022, this university employed a traditional teaching model, relying primarily on classroom lectures and face-to-face teacher-student interactions. However, beginning in September 2022, in line with new policies from the Ministry of Education of China, the university actively promoted a transition to digital teaching, gradually integrating digital technologies into the medical education process. Consequently, our study cohort includes students who experienced both traditional teaching and the transition to digital teaching environments.

The study sample comprised 529 medical students and the research spanned a 12-month period from February 2022 to January 2023. During this time, students were exposed to traditional teaching in the first semester (February to August 2022) and to digital teaching in the second semester (September 2022 to January 2023). Data were collected through a questionnaire follow-up survey every two months; this survey was designed to capture changes in key variables, including learning burnout, self-efficacy and learning adaptability.

In the sample design, potential confounding factors, including gender, age, academic background, and performance, were carefully controlled to ensure the representativeness and scientific rigor of the data. For instance, gender and age are established factors influencing stress coping strategies and psychological maturity [42, 43], while socioeconomic background factors such as parental education level are known to correlate with students’ academic adaptation and mental health outcomes [42, 44]. Firstyear students were chosen as the research focus because they are in a critical phase of adapting to university life and new teaching models, making them an ideal group for investigating the impact of digital teaching on learning burnout. This design not only allowed us to analyze the overall effects of digital teaching on learning burnout but also enabled us to explore the mediating and moderating roles of selfefficacy and learning adaptability.

### Inclusion and exclusion criteria

#### Inclusion criteria

1. Enrollment Status: Participants needed to be first-year medical students enrolled at a high-level university in Guangdong Province, China, with a complete on-campus study experience during the research period.
2. Teaching Mode Experience: Students needed to have experienced traditional teaching from February 2022 to August 2022 and transitioned to digital teaching from September 2022 to January 2023, thus ensuring they fully experienced the shift between the two teaching modes.
3. Willingness to Participate: Students needed to participate voluntarily in this study, sign an informed consent form, fully understand the purpose and procedures of the study, and agree to a 12-month tracking survey.
4. Data Completeness: Participants needed to complete all data collection tasks throughout the study to ensure data continuity and integrity, including monthly questionnaire surveys.
5. Psychological and Behavioral Competence: Students needed to possess the basic ability to read and complete questionnaires independently, including those that measured learning burnout, self-efficacy and learning adaptability.

#### Exclusion criteria

1. Interruption of Enrollment: Students who suspended their studies, transferred to another institution, or were otherwise unable to complete the full study tasks during the research period were excluded.
2. Non-compliance with Teaching Experience: Students who did not fully participate in the digital teaching reform beginning in September 2022, such as those missing significant portions of the coursework or were frequently absent, were excluded.
3. Data Absence: Students missing significant data related to key variables, caused by either subjective or objective reasons (e.g., failure to submit questionnaires or incomplete responses), were excluded.
4. Special Health Conditions: Students with severe psychological disorders or cognitive impairments that prevented them from completing the questionnaires or experimental procedures were excluded.

**Fig. 1:**
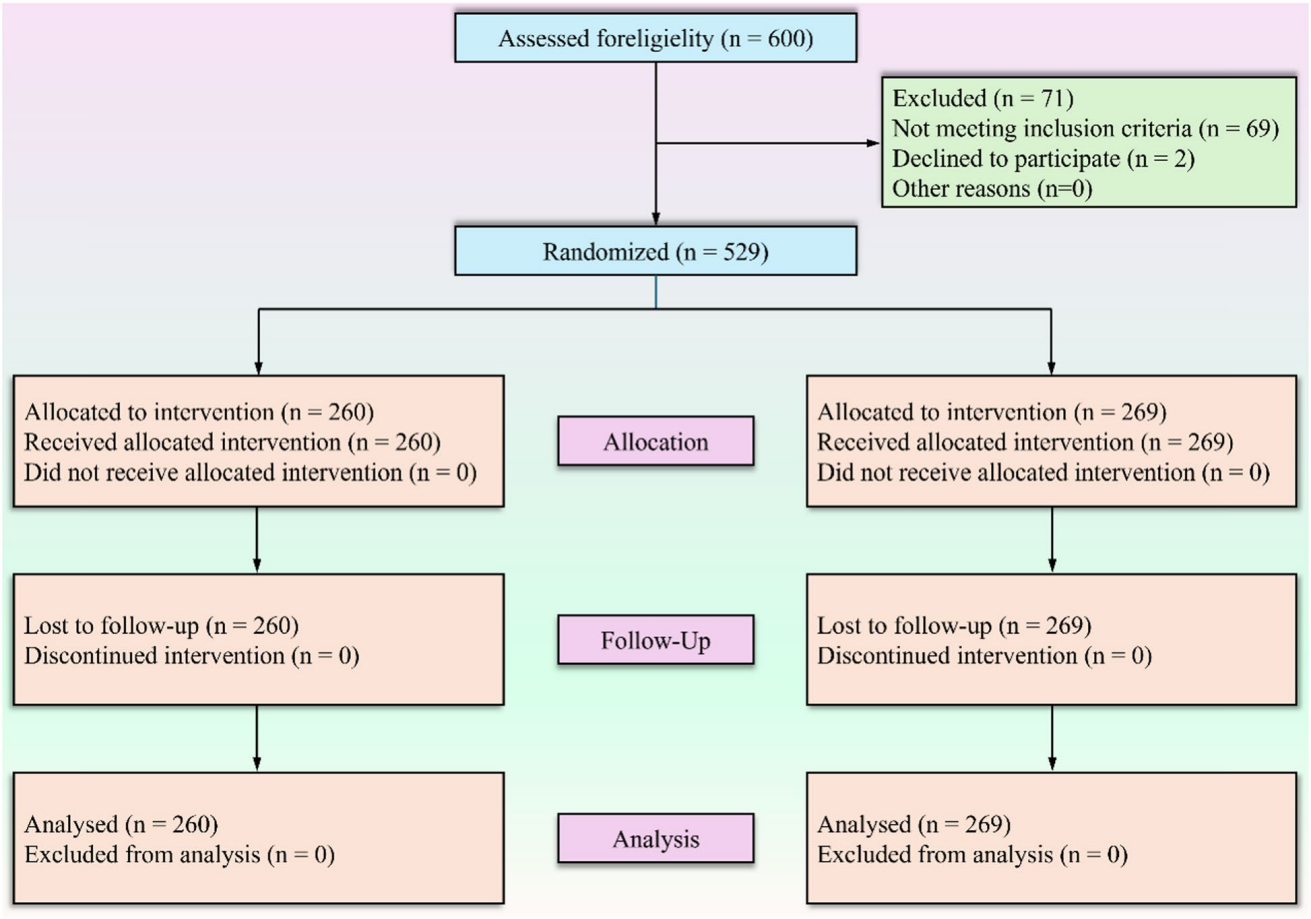
CONSORT flowchart for this study showing the specific inclusion and exclusion criteria

In summary, the tracking survey initially targeted 600 students. After applying the inclusion and exclusion criteria, valid data from 529 students were retained for analysis (Fig. 1).

### The selection of main variables and tools

#### College students’ learning burnout (CSLB)

CSLB refers to the negative emotions and behaviors exhibited by students during the learning process due to prolonged pressure, a lack of motivation, and emotional exhaustion. CSLB is characterized by emotional fatigue, a lack of a sense of achievement, and reduced learning motivation, which impacts both academic performance and mental health [45]. This study employed the CSLB Scale developed by Lian et al. [46], which includes three dimensions: a low sense of achievement, emotional exhaustion, and inappropriate behavior (supplementary file). This scale consists of 20 items rated on a 5-point Likert scale (1 = “completely disagree,” 5 = “completely agree”), with eight items been scored in a reverse manner. The total score reflects the level of learning burnout, with higher scores indicating more severe burnout. In this study, Cronbach’s α coefficient for the scale was 0.784, indicating good internal consistency.

#### Digital teaching (DT)

DT refers to the use of information technologies, particularly the internet and digital tools, to support and enhance the teaching process [47]. This teaching approach overcomes the time and space limitations of traditional classrooms, allowing students to engage in autonomous learning and interactions via online platforms and virtual classrooms. Digital teaching is characterized by flexibility, efficiency, and personalization, offering tailored learning content based on the real needs of students and enhancing engagement via online discussions and real-time feedback [48]. However, excessive reliance on self-learning and screen-based study, along with reduced face-to-face interaction, may lead to reduced motivation and increased psychological stress [49]. In the present study, we considered digital teaching as a binary variable to investigate its effect on learning burnout. Students who did not receive digital teaching were coded as 0, while those who did were coded as 1.

#### Self-efficacy (SE)

Self-efficacy refers to an individual’s confidence and perceived ability to successfully complete specific tasks or overcome challenges [50]. In this study, we utilized the General Self-Efficacy Scale (GSES) developed by Schwarzer et al. [32], which assesses an individual’s confidence in handling various life tasks and coping with stress (supplementary file). The scale consists of 10 items that are rated on a 4-point Likert scale (1 = “not at all true,” 4 = “exactly true”), with a total score of 40. Higher scores indicate stronger levels of self-efficacy. In this study, Cronbach’s α coefficient for the scale was 0.890, demonstrating excellent internal consistency and reliability.

#### Learning adaptability (LA)

Learning adaptability refers to the ability of students to adjust their state and cope with learning tasks in different educational environments; this directly impacts their academic performance and mental health. In this study, we used the Learning Adaptability Scale developed by Feng et al. [51] based on a conceptual model of learning adaptability. The scale includes five dimensions: learning motivation, teaching mode adaptability, learning ability, learning attitude, and environmental factors and uses a 5-point Likert scale, with higher scores indicating stronger levels of adaptability (supplementary file).

#### Control variables

In this study, we controlled for other factors that may have influenced the experiment. Gender was coded as 1 for male and 2 for female. Household registration type was coded as 1 for rural and 2 for urban. Ethnicity was coded as 1 for Han and 2 for other ethnic minorities. For being one-child, multi-child were coded as 1, and one-child as 2. Parental education levels were coded as 1 for junior high school, 2 for senior high school, 3 for undergraduate, and 4 for graduate. In addition, student age was included as a control variable.

#### Research tools and methods

In this study, we primarily utilized Stata 17 software (Stata Corporation, College Station, TX) for data analysis. Prior to September 2022, students were taught using traditional methods. After September 2022, Guangdong Medical University actively integrated digital technology into education in response to the Ministry of Education’s requirements, marking the transition to digital teaching. Therefore, September 2022 served as a critical cutoff point in this study. The specific regression model utilized in this study is described by Eqs. (1, 2, 3, 4).

1 $${\mathrm{CSLB}}_{\mathrm i,\mathrm t}=\mathrm\alpha+{\mathrm\beta}_1{\mathrm D}_{\mathrm i,\mathrm t}+\gamma\mathrm f\left({\mathrm D}_{\mathrm{it},\;}\mathrm x\right)++{\mathrm\beta}_2\mathrm{Controls}++{\mathrm\varepsilon}_{\mathrm i,\mathrm t}$$

In Eq. (1), ‘CSLB’ represents the learning burnout of students; ‘x’represents the driving variable indicating the number of survey intervals since the introduction of digital teaching; ‘Control’ represents the control variables; and ‘_i, t_‘denotes the error terms.

During the study, the moderation effect analysis model was described by Eq. (2).

2 $${\mathrm{CSLB}}_{\mathrm i,\mathrm t}=\mathrm\alpha+{\mathrm\beta}_1{\mathrm{DT}}_{\mathrm i,\mathrm t}+{\mathrm\beta}_1\mathrm{SE}\ast{\mathrm{DT}}_{\mathrm i,\mathrm t}+\gamma\mathrm{Controls}++{\mathrm\varepsilon}_{\mathrm i,\mathrm t}$$

In Eq. (2), ‘SE’ represents the moderating variable self-efficacy, while ‘SE*DT’represents the interaction term between self-efficacy and digital teaching. Other variables are defined in Eq. (1).

The mediation effect analysis models are given by Eqs. (3) and (4).

3 $${\mathrm{LA}}_{\mathrm i,\mathrm t}=\mathrm\alpha+{\mathrm\beta}_1{\mathrm{DT}}_{\mathrm i,\mathrm t}+\gamma\mathrm{Controls}+{\mathrm\varepsilon}_{\mathrm i,\mathrm t}$$

4 $${\mathrm{CSLB}}_{\mathrm i,\mathrm t}=\mathrm\alpha+{\mathrm\beta}_1{\mathrm{DT}}_{\mathrm i,\mathrm t}+\mathrm\beta2{\mathrm{LA}}_{\mathrm i,\mathrm t}+\gamma\mathrm{Controls}+{\mathrm\varepsilon}_{\mathrm i,\mathrm t}$$

In Eqs. (1) and (2), ‘LA’ represents the mediating variable ‘learning adaptability’. All other variables are as previously defined.

### Experimental intervention

In this experiment, we defined September 2022 as the critical time point, introducing digital teaching as an intervention to track and analyze changes in learning state between traditional and digital teaching modes. The intervention process was divided into three main phases: preparation, implementation and follow-up monitoring.

#### Preparation phase

Between February and August 2022, students were engaged in traditional teaching, which primarily relied on classroom lectures. Teachers delivered knowledge using blackboards and PowerPoint presentations, while students took notes and participated in group discussions during class. During this phase, the research team conducted baseline measurements to collect data relating to learning burnout, self-efficacy, and learning adaptability, thus providing comparative data for the evaluation of intervention effects. In addition, we developed a detailed intervention plan to ensure the effective implementation of digital teaching. This included training teachers in digital teaching skills, constructing digital resources, and setting up an online learning platform.

#### Implementation phase

Commencing in September 2022, Guangdong Medical University officially introduced the digital teaching model. This approach transformed traditional teaching methods by leveraging online learning platforms, virtual classrooms, digital resource sharing, and intelligent teaching tools. Students accessed course materials, participated in real-time interactive classes, and completed online tests and assignments through the university’s online platform. During the intervention, the research team regularly monitored the learning behavior of students, including time allocation, participation in online courses, and the frequency of using teaching resources. To ensure teaching quality, the research team collaborated with teachers to optimize course content and adjust teaching strategies, thus helping students adapt to the digital teaching model.

#### Follow-up monitoring phase

After implementing digital teaching, the research team conducted monthly measurements of learning burnout, self-efficacy, and learning adaptability among the study cohort for four months. This long-term tracking allowed us to capture changes in the psychological states and behavioral patterns of students before and after the intervention. Throughout this intervention process, we not only investigated the impact of digital teaching on learning burnout but also analyzed the mediating and moderating roles of self-efficacy and learning adaptability in this process. These findings provided empirical support for improving teaching effectiveness and promoting the mental health of students.

## Results

### Descriptive statistical analysis

To compare changes in CSLB, self-efficacy (SE), and LA before and after the digital teaching experiment, we divided the eight measurement results into a traditional teaching group (Measurements 1–4) and a digital teaching group (Measurements 5–8) for comparison (Table 1). In order to achieve homogeneity and consistency in the distribution of students at different levels of education, we compare the demographic and background characteristics of the students in the study. In terms of student age, there were 162 students aged 18years, 171 students aged 19 years, and 196 students aged 20 years. There are 310 male students and 219 female students. Male students represented the majority (58.6%), while females made up 41.4%. With regard to the Parents’ Highest Level of Education (PHLE), the parents of 151 students had parents for whom the highest level of education was junior high school, the parents of 123 students had received high school education, the parents of 133 had an undergraduate degree, and the parents of 122 students had a graduate school degree (Table 2). This distribution indicates a fairly even spread across educational levels.

**Table 1:** The performance of students in traditional teaching and digital teaching

Group	Variable	*N*	Mean	p50	SD	Min	Max
Traditional teaching	CSLB	529	40.127	40	6.167	30	50
	SE	529	35.268	35	7.429	23	48
	LA	529	20.493	20	2.331	17	24
Digital teaching	CSLB	529	67.327	67	13.273	45	90
	SE	529	35.282	35	7.565	23	48
	LA	529	12.495	12	4.563	5	20

*CSLB* College atudents’ learning burnout, *SE* Self-efficacy, *LA* Learning adaptability

**Table 2:** Demographic and background characteristics of the student participants (*N* = 529)

Variable	Category	Number of students	%
Age	18	162	30.6
	19	171	32.3
	20	196	37.1
Gender	Male	310	58.6
	Female	219	41.4
Ethnicity	Han ethnic group	252	47.6
	Others	277	52.4
Residential background	1	259	49.0
	2	270	51.0
One-child	No	273	51.6
	Yes	256	48.4
Highest level of parental education	Junior high school	151	28.5
	High School	123	23.3
	Undergraduate	133	25.1
	Graduate School	122	23.1
Academic Year	First-year	529	100

Residential background, 1 = rural, 2 = urban

With regards to CSLB and traditional teaching, the mean CSLB and standard deviation (SD) was 40.127 ± 6.167, with a median (p50) of 40, indicating relatively low variability, with a range of 30–50. For digital teaching, the mean CSLB and SD was 67.327 ± 13.273, with a median of 67, thus showing higher variability than traditional teaching, with a range of 45–90. Students in the digital teaching group exhibited higher levels of learning burnout compared to those in the traditional teaching group (0.040), thus suggesting a potential negative impact of digital teaching on learning burnout.

With regards to SE, for traditional teaching, the mean SE and SD was 35.268 ± 7.429, with a median of 35, and a range of 23–48. For digital teaching, the mean SE and SD was 35.282 ± 7.565, with a median of 35. The range was identical to traditional teaching, (23–48). There was no significant difference in self-efficacy between the two teaching groups.

With regards to LA, for traditional teaching, the mean LA and SD was 20.49 ± 2.331, with a median of 20, indicating low variability, with a range of 17–24. For digital teaching, the mean LA and SD was 12.495 ± 4.563, with a median of 12 and ranging from 5 to 20. Students in the digital teaching group demonstrated significantly lower learning adaptability compared to those in the traditional teaching group (0.015), thus suggesting that digital teaching might pose challenges to adapting to learning processes.

### Main effects analysis

To determine the impact of digital teaching adoption on learning burnout in university students, we employed regression analysis to investigate the causal relationship between these two parameters (Table 3). Without adding control variables, the regression coefficient for the effect of digital teaching on learning burnout was 0.034, indicating a statistically significant positive correlation. When control variables were included, the regression coefficient increased slightly to 0.042 (*P* < 0.001), further confirming the robustness of the relationship. Furthermore, the inclusion of control variables, such as age, gender, academic year, and parental education, is known to enhance the reliability of the results.

**Table 3:** The impact of digital teaching adoption on learning burnout in university students

Targets	CSLB (1)	CSLB (2)
DT	0.034^***^	0.042^***^
	(4.13)	(5.06)
Age		0.567
		(0.88)
Gender		−0.296
		(−0.28)
Ethnicity		0.00349
		(0.00)
Account		0.653
		(0.62)
One-child		−0.116
		(−0.11)
PHLE		−0.310
		(−0.66)
_cons	53.73^***^	43.28^***^
	(102.24)	(3.46)
*R* ^2^	0.001	0.001
adj. *R*^2^	0.001	0.004

*t* statistics in parentheses

*CSLB* College students’ learning burnout, *DT* Digital teaching, *PHLE* Parents’ Highest Level of Education

^*^P < 0.1, ^**^P < 0.05, ^***^P < 0.01

### The mediating role of learning adaptability

To further investigate the mediating role of learning adaptability, we used the PROCESS macro with Model 1 to examine the learning adaptability mediating effect through biologically calibrated self-guided sampling (5000 iterations) [52]. We first measured the impact of digital teaching on learning adaptability (Model 1 in Table 4) and then assessed the combined effects of digital teaching and learning adaptability on learning burnout in university students (Model 2 in Table 4). Analysis revealed a regression coefficient of 0.015 between digital teaching and learning adaptability, thus indicating that the adoption of digital teaching can significantly enhance learning adaptability. Furthermore, the mediating effect coefficient of learning adaptability in the relationship between digital teaching and learning burnout was − 1.835 (*P* < 0.001). This finding suggests that, although digital teaching had a positive direct impact on learning burnout, it indirectly reduced learning burnout by improving learning adaptability.

**Table 4:** The mediating effect of learning adaptability and the moderating effect of self-efficacy

Targets	LA	CSLB	CSLB
DT	0.015^**^	0.040^***^	
	(2.81)	(4.73)	
Age	0.114	0.776	0.567
	(0.56)	(1.48)	(0.88)
Gender	0.413	0.461	−0.296
	(1.22)	(0.53)	(−0.28)
Ethnicity	0.00000929	0.00351	0.00349
	(0.00)	(0.00)	(0.00)
Account	0.279	1.165	0.653
	(0.84)	(1.35)	(0.62)
One-child	−0.287	−0.642	−0.116
	(−0.86)	(−0.74)	(−0.11)
PHLE	0.0713	−0.179	−0.310
	(0.48)	(−0.47)	(−0.66)
LA		−1.835^***^	
		(−23.00)	
DT*SE			−0.062^**^
			(2.18)
_cons	13.56^***^	68.17^***^	43.28^***^
	(3.44)	(6.64)	(3.46)
*R* ^2^	0.004	0.336	0.001
adj. *R*^2^	0.002	0.332	0.004

*t* statistics in parentheses

*CSLB* College atudents’ learning burnout, *SE* Self-efficacy, *LA* Learning adaptability, *DT* Digital teaching, *PHLE* Parents’ Highest Level of Education

^*^P < 0.1, ^**^P < 0.05, ^***^P < 0.01

### The moderating role of self-efficacy

To examine the mediating role of learning adaptability between digital teaching and learning burnout, we used the PROCESS macro with Model 1 to test mediation effects via biascorrected bootstrap sampling [52]. We investigated the moderating effect of self-efficacy on the relationship between digital teaching and learning burnout in university students by constructing an interaction term between digital teaching and self-efficacy (DT*SE). The regression coefficient for the interaction term (DT*SE) and learning burnout was − 0.062 (*P* < 0.05) (Table 4). This indicates that self-efficacy significantly moderated the impact of digital teaching on learning burnout.

## Discussion

### The impact of digital teaching on learning burnout and the mediating role of learning adaptability

The findings of this study demonstrate a significant positive relationship between digital teaching and learning burnout in university students; this is consistent with prior research highlighting the challenges of digital learning environments [35]. Digital teaching, while providing flexibility and access to diverse resources, often increases cognitive overload and reduces direct interpersonal interactions, leading to enhanced levels of stress and fatigue. Our present findings add to the existing literature by quantitatively confirming this relationship, with regression coefficients indicating a significant increase in learning burnout associated with digital teaching. However, the mediating role of learning adaptability provides a nuanced understanding of this relationship. Our results show that while digital teaching leads to increased levels of learning burnout, it simultaneously enhances learning adaptability, which can partially mitigate burnout. This proactive personal adjustment behavior indirectly alleviates burnout. It reflects the complex interplay between environmental, behavioral, and personal factors. The significant mediating effect we observed (regression coefficient: −1.835) suggests that improving the adaptability of students by digital literacy training and providing structured support mechanisms could serve as a protective factor against burnout.

Our findings highlight the need for universities to adopt a dual approach to digital teaching. First, institutions should design digital teaching strategies that minimize burnout by reducing cognitive overload. This could include breaking down content into manageable segments, providing clearer instructions, and by incorporating frequent feedback mechanisms. Second, fostering learning adaptability should become a priority in digital education. Universities could introduce training programs that enhance the digital literacy, time management, and problem-solving skills of students. Furthermore, incorporating adaptability-focused pedagogical practices, such as scenario-based learning and peer collaboration, could further enhance the capacity of students to thrive in digital environments. By aligning teaching methods with the adaptability levels of students, institutions could maximize the benefits of digital teaching while mitigating its drawbacks.

### The moderating role of self-efficacy on the relationship between digital teaching and burnout

Our findings related to the moderating role of self-efficacy add an important dimension to our understanding of the dynamics of digital teaching and burnout. Interaction analysis revealed that students with higher self-efficacy were less affected by the burnout-inducing aspects of digital teaching when compared to those with lower self-efficacy. This finding aligns with the theory put forward by Bandura et al. [11] which posited that self-efficacy influences how individuals perceive challenges and their ability to overcome them. In the context of digital teaching, students with high levels of self-efficacy are more confident in navigating complex digital tools, managing self-directed learning, and overcoming obstacles, thus experiencing less burnout. Conversely, students with low levels of self-efficacy may feel overwhelmed by the increased autonomy and responsibility required in digital learning environments. These students are more likely to perceive the challenges as insurmountable, leading to frustration and exhaustion. The moderating effect identified in our current study suggests that self-efficacy acts as a psychological resource that buffers the negative impact of digital teaching on learning burnout.

To reduce burnout, educational institutions should prioritize strategies that can enhance the self-efficacy of students. This can be achieved through interventions such as structured goal-setting, where students can be encouraged to set realistic and achievable milestones in their learning journey. Providing personalized feedback that highlights individual progress and accomplishments can also boost confidence. Furthermore, peer mentoring programs can be introduced to allow students with high levels of self-efficacy to guide their peers, thus creating a supportive learning environment. From the perspective of curriculum design, the incorporation of small and success-oriented tasks in digital teaching could help build a sense of competence among students, especially those with low levels of self-efficacy. For example, gamification elements, such as badges, points, and rewards for completing tasks could provide positive reinforcement and motivate students. These strategies not only reduce burnout but also foster a growth mindset, thus enabling students to view challenges as opportunities for development rather than threats.

### The dual impact of digital teaching and learning on students

Digital teaching has a double impact on students. On one hand, the shift to digital teaching may lead to higher levels of learning burnout due to factors such as increased screen time, reduced social interactions and cognitive overload. On the other hand, digital teaching also facilitates opportunities for personalized and flexible learning, enabling students to develop better adaptability to new learning environments and demands. The significant mediating role of learning adaptability implies that students who can adjust effectively to digital teaching environments are less likely to experience burnout. This finding has important implications for educational institutions. To mitigate the potential negative effects of digital teaching on mental health, universities should focus on fostering learning adaptability. This could include offering structured guidance, providing digital literacy training, and creating supportive online learning communities to help students navigate digital learning platforms more effectively. By enhancing student adaptability, universities can leverage the benefits of digital teaching while minimizing its drawbacks, ultimately promoting a healthier and more effective learning environment.

### Limitations

Although this study provides valuable insights, certain limitations must be acknowledged. First, our study cohort was drawn from a single university in Guangdong Province, thus limiting the generalizability of our findings. The experience of students with digital teaching can vary widely based on regional, cultural and institutional differences. For instance, students in institutions with an advanced technological infrastructure may experience fewer challenges when compared to those in resource-constrained settings. Future research should expand the study cohort to include diverse regions and educational contexts; this would enhance the external validity of our findings. Second, the reliance on self-reported measures introduces the possibility of bias relating to social desirability. While the validated scales used in this study ensured reliability, the subjective perceptions of students may not fully capture the objective reality of their experiences. The incorporation of additional data sources, such as behavioral analytics from learning management systems or teacher evaluations, could provide a more comprehensive understanding of the digital teaching-burnout dynamic. Finally, the cross-sectional design of our study limits our ability to draw causal inferences. While the regression models and interaction analyses provide robust evidence, a longitudinal study would allow researchers to track changes in learning burnout, adaptability and self-efficacy over time, thus offering deeper insights into their dynamic interplay. Despite these limitations, our findings provide Modular curriculum and incremental instructional task design for researchers and practitioners. Future studies could investigate the long-term effects of digital teaching on student outcomes by employing longitudinal designs. Furthermore, integrating mixed-method approaches, such as qualitative interviews or focus groups, could enrich our understanding of how students perceive digital teaching. Researchers could also investigate the role of external factors, such as family support and access to technology; these factors could interact with digital teaching to influence learning burnout.

## Conclusions

In this study, we comprehensively investigated the impact of digital teaching on learning burnout in university students, and the roles of self-efficacy and learning adaptability as moderating and mediating factors, respectively. Our findings provide valuable insights into the intricate dynamics of digital education and its psychological effects on students. First, we identified a significant positive relationship between digital teaching and learning burnout. Digital teaching, while offering flexibility and accessibility, often increases cognitive demands, reduces interpersonal interaction, and introduces technical challenges, all of which contribute to enhanced levels of burnout. However, learning adaptability plays a crucial mediating role in mitigating these effects. Students with higher levels of adaptability can adjust their learning strategies and attitudes to better cope with the demands of digital education, thus reducing the overall impact of burnout. This underscores the importance of fostering adaptability through targeted interventions such as digital literacy training, time management workshops and collaborative learning environments. These initiatives can empower students to navigate digital platforms effectively and maximize the potential benefits of digital teaching while minimizing its drawbacks. Second, our findings highlight the protective role of self-efficacy in the relationship between digital teaching and burnout. These findings indicate that self-efficacy moderates this relationship, with students possessing higher levels of self-efficacy experiencing less burnout than their peers with lower self-efficacy. This suggests that confidence in one’s ability to succeed in digital learning contexts serves as a buffer against stress and frustration. Practical implications include designing educational programs that build self-efficacy through personalized feedback, structured goal-setting and peer mentoring. These approaches could help students to develop resilience and a growth mindset, thus enabling them to view challenges as opportunities for skill development rather than insurmountable barriers. Collectively, our findings contribute to the growing body of literature relating to digital education by elucidating the complex interplay between digital teaching, learning burnout, self-efficacy and learning adaptability. Our findings have significant implications for educators and policymakers, providing actionable strategies to improve digital learning outcomes and support the mental health of students. By prioritizing the development of adaptability and self-efficacy, educational institutions can create more effective and sustainable digital teaching frameworks, thus ensuring that students thrive in an increasingly technology-driven academic landscape.

## Supplementary Information

Supplementary Material 1.

## Data Availability

All data generated during this study are included in the paper. Further information can be obtained from the corresponding author.

## Abbreviations

CSLB: College Students’ Learning Burnout
DT: Digital Teaching
SE: Self-efficacy
GSES: General Self-Efficacy Scale
LA: Learning Adaptability
PHLE: Parents’ Highest Level of Education
