# Supplementary material for: The impact of digital teaching on learning burnout in college students: the moderating role of self-efficacy and the mediating effect of learning adaptability
Source: BMC Med Educ. 2026 Jan 7;26:194. doi: 10.1186/s12909-025-08330-0 (PMC12870430; doi:10.1186/s12909-025-08330-0)
Supplement: Supplementary file 1 — Supplementary Material 1. [file 12909_2025_8330_MOESM1_ESM.docx]

**College Student Learning Burnout Scale (Revised)**

Modified based on the literature: Rong Lian, Lixian Yang, Lanhua Wu et al., 2005

**Dimensions:**

1. Low Mood (8 items)

2. Inappropriate Behavior (6 items)

3. Low Sense of Achievement (6 items)

Reliability and Validity:

Cronbach’s α coefficient: 0.865

Subscaletotal correlations:

Low Mood: 0.914

Inappropriate Behavior: 0.799

Low Sense of Achievement: 0.704 (all \( p < 0.01 \))

5point Likert scale:

⑤ Fully agree → ① Completely disagree

Reverse scoring applies to items marked with an asterisk ().

**Scale Items**

1. I have my own learning methods and plans and can implement them effectively. ⑤④③②①

2. I think the knowledge I learned is useless. ⑤④③②①

3. I find it easy to master professional knowledge. ⑤④③②①

4. I feel exhausted when getting up early in the morning. ⑤④③②①

5. I struggle to maintain passion when facing a day of study. ⑤④③②①

6. I can calmly handle emotional issues during study. ⑤④③②①

7. After a day of study, I feel physically drained. ⑤④③②①

8. My university studies have fully demonstrated my abilities. ⑤④③②①

9. I am tired of studying. ⑤④③②①

10. I rarely engage in studying. ⑤④③②①

11. I can manage university courses effectively. ⑤④③②①

12. I often doze off while studying. ⑤④③②①

13. I am highly interested in my major. ⑤④③②①

14. I lack patience in studying. ⑤④③②①

15. Obtaining a bachelor’s degree is easy for me. ⑤④③②①

16. I only study when exams approach. ⑤④③②①

17. I want to study but find it boring. ⑤④③②①

18. I feel energetic while studying. ⑤④③②①

19. I seldom plan my study time. ⑤④③②①

20. Exams always annoy me. ⑤④③②①

Notes: Reverse scored items: 1, 3, 6, 8, 11, 13, 15, 18 (marked with).

**Scoring Rules**

1. Factor Scores:

Low Mood: Sum of items 2, 4, 5, 7, 9, 12, 17, 20.

Inappropriate Behavior: Sum of items 1, 8, 10, 14, 16, 19.

Low Sense of Achievement: Sum of items 3, 6, 11, 13, 15, 18.

2. Total Score = Sum of all three factor scores.

Range: 20–100.

Higher scores indicate greater learning burnout.

**References**

1. Lian, R., Yang, L., & Wu, L. (2005). Development and validation of the College Student Learning Burnout Scale. Journal of Educational Psychology, 27(3), 123135.

2. College Student Mental Health Guidance Center. (n.d.). College Student Learning Burnout Scale (LBUS). Retrieved from https://zxzx.xxu.edu.cn/info/1985/1623.htm

Note: Ensure consistency in item numbering and reverse scoring during administration. For research use, validate the scale within the target population.

**College Students' Learning Adaptation Scale (LAS)**

**Developers and Background**

The Learning Adjustment Scale (LAS) was developed by Chinese scholars Tingyong Feng, Ti Su, Xingwang Hu, and Hong Li in 2006. This scale is specifically designed for undergraduate students in China and has been culturally adapted to reflect the educational context of Chinese universities.

**Scale Structure**

The LAS comprises 5 dimensions measured through 29 items, utilizing a 5-point Likert scale (1 = Strongly Disagree to 5 = Strongly Agree). Higher total scores indicate better learning adaptation.

**Dimensions and Sample Items**

1. Learning Motivation (8 items)

- I have developed clear learning goals since entering university.

- I implement personalized study methods and plans effectively.

- I feel a strong sense of urgency to acquire knowledge.

- Reverse-scored item: I have become noticeably less diligent after starting college.

2. Adaptability to Teaching Methods (7 items)

- I adapt well to university instructors' teaching styles.

- I manage my schedule efficiently in the university environment.

- Reverse-scored item: I struggle with the transition from high school to university teaching methods.

3. Learning Ability (6 items)

- I proactively seek help when encountering academic challenges.

- I efficiently organize study time to improve productivity.

- Reverse-scored item: I find university courses overwhelmingly difficult.

4. Learning Attitude (4 items)

- I maintain a strong intrinsic interest in learning.

- I regard academic achievement as a high priority.

5. Environmental Factors (4 items)

- The campus learning atmosphere strongly supports my studies.

- Harmonious peer relationships enhance my academic performance.

**Scoring and Interpretation**

- Total Score Range: 29–145 (sum of all item scores).

- Dimension Analysis: Sub-scores for each dimension help identify specific adaptation strengths or weaknesses.

- Reverse-Scored Items: 6 items (e.g., "I have become noticeably less diligent") require inverse coding (1→5, 2→4, etc.).

**References**

1. Feng, T., Su, T., Hu, X., & Li, H. (2006). Development and validation of the College Students' Learning Adaptation Scale. Journal of Psychology, 38(5), 652-660.

2. Schwarzer, R., & Jerusalem, M. (1995). Generalized Self-Efficacy scale. In J. Weinman et al. (Eds.), Measures in Health Psychology (pp. 35-37). NFER-NELSON.

**General Self-Efficacy Scale (GSES)**

**Developers and Background**

The General Self-Efficacy Scale (GSES) was initially developed in 1981 by Professor Ralf Schwarzer and Matthias Jerusalem at the Free University of Berlin, Germany. The original version contained 20 items, which was later refined to a 10-item scale through empirical validation.

**Scale Format and Scoring**

- The GSES uses a 4-point Likert scale for each item:

1 = Completely incorrect

2 = Partially incorrect

3 = Partially correct

4 = Completely correct

- Total scores range from 10 to 40, with higher scores indicating stronger self-efficacy.

**10 Items of the GSES**

1. I believe I can cope with all kinds of difficulties.

2. I can stick to my ideas and work hard to achieve my goals when faced with difficulties.

3. I still have a way to get what I want even if others oppose me.

4. I can deal with all kinds of unexpected situations.

5. I can effectively handle various challenges.

6. I can solve most of the problems I encounter.

7. I can face difficulties calmly because I believe in my ability to handle problems.

8. As long as I work hard, I can achieve what I want.

9. I believe I can manage any emergency.

10. I can stay calm in difficult situations.

**Reference**

Schwarzer, R., & Jerusalem, M. (1995). Generalized Self-Efficacy scale. In J. Weinman, S. Wright, & M. Johnston (Eds.), Measures in health psychology: A user’s portfolio. Causal and control beliefs (pp. 35-37). Windsor, UK: NFER-NELSON.
